# Chaperonin Structure – The Large Multi-Subunit Protein Complex

**DOI:** 10.3390/ijms10030844

**Published:** 2009-03-02

**Authors:** Mateusz Banach, Katarzyna Stąpor, Irena Roterman

**Affiliations:** 1 Department of Bioinformatics and Telemedicine – Jagiellonian University, Collegium Medicum, Lazarza 16, 31-531 Krakow, Poland; E-Mail: myroterm@cyf-kr.edu.pl; 2 Faculty of Physics, Astronomy and Applied Computer Science - Jagiellonian University, Reymonta 4, 30-059 Krakow, Poland; E-Mail: mateusz.banach@uj.edu.pl; 3 Silesian Technical University, Institute of Computer Science, Akademicka 16 44-100 Gliwice, Poland; E-Mail: katarzyna.stapor@polsl.pl

**Keywords:** Protein folding, hydrophobicity, chaperonin

## Abstract

The multi sub-unit protein structure representing the chaperonins group is analyzed with respect to its hydrophobicity distribution. The proteins of this group assist protein folding supported by ATP. The specific axial symmetry GroEL structure (two rings of seven units stacked back to back - 524 aa each) and the GroES (single ring of seven units - 97 aa each) polypeptide chains are analyzed using the hydrophobicity distribution expressed as excess/deficiency all over the molecule to search for structure-to-function relationships. The empirically observed distribution of hydrophobic residues is confronted with the theoretical one representing the idealized hydrophobic core with hydrophilic residues exposure on the surface. The observed discrepancy between these two distributions seems to be aim-oriented, determining the structure-to-function relation. The hydrophobic force field structure generated by the chaperonin capsule is presented. Its possible influence on substrate folding is suggested.

## 1. Introduction

It has been discovered that the protein folding process is guided by additional molecules directing the structural changes toward the correct native form. Molecular chaperones are the proteins which bind and stabilize unfolded or partially folded proteins, thereby preventing them from being degraded [1–4]. Only a certain subset of cellular proteins undergo the folding process accompanied by the chaperonins, which are large protein constructs which directly facilitate the protein folding process with participation of ATP molecules. Chaperonin exists as a back-to-back linked double-ring complex. The symmetric (7-fold) rings of GroEL interact with the co-chaperonin GroES. The mechanism of ATP binding and its collaboration with internal structural changes in *cis-* (called chains A-G in this paper) and *trans-*rings (chains H-N in this paper) reveals the functioning algorithm of the folding machine. Each part is responsible for a specific element of this algorithm [1–4].

The object of the analysis was the chaperonin used as an example to search for possible mechanisms for the generation of such large constructions with a nano-machine character. The question may be asked, how do these proteins become folded? How do they influence the substrate folding?

An attempt to find answers to these questions on the basis of the “fuzzy oil drop” model [5–13] has been undertaken and is presented in this paper. The assumed model helps clarify to what extent the hydrophobicity distribution may help, support or even direct the protein folding. The specific role of chaperonins as nano-machines with two functions: first as “holder” – complexation of the folding molecule to prevent the misfolding and secondly, as a “folder” – directing the folding process to the generation of proper native structure is the object of the work presented [14].

The structure of the GroEL-GroES-(ADP)7 complex is a very good example to test the “fuzzy-oil-drop” model applicability to recognize the structural and functional specificity of the protein under consideration due to multi-subunit protein assembly comprising rings of subunits stacked back to back. The presence of ADP ligands makes possible the analysis of ligand docking to this molecule. The complete complex is presented in parts distinguishing the structural and functional fragments of this multi-subunit construction.

## 2. Materials and Methods

### 2.1. Data

The 1AON – the structure of the object under consideration has been taken from the PDB (deposit 1AON) [15].

### 2.2. “Fuzzy-oil-drop” Model

It is assumed that the presence of an external force field of hydrophobic character expressed by the three-dimensional Gauss function is able to direct the protein folding toward hydrophobic core generation, with the simultaneous exposure of hydrophilic residues toward the surface of the protein molecule.

The external force field is represented by the three-dimensional Gauss function. The value of the Gauss function (traditionally interpreted as probability density value) is assumed to represent the hydrophobic density in the protein body. The hydrophobicity density can be calculated for any point the space covering the protein molecule.

The three-dimensional Gauss function is given as follows:

$$Ht_{j}=\frac{1}{Ht_{sum}}\;\text{exp}\left(\frac{-{\left(x_{j}-\bar{x}\right)}^{2}}{2\sigma_{x}^{2}}\right)\;\text{exp}\left(\frac{-{\left(y_{j}-\bar{y}\right)}^{2}}{2\sigma_{y}^{2}}\right)\;\text{exp}\left(\frac{-{\left(z_{j}-\bar{z}\right)}^{2}}{2\sigma_{z}^{2}}\right)$$

The value of *Ht_j_* is assumed to represent the hydrophobicity distribution at a particular point belonging to the protein body. The hydrophobicity maximum is localized in the center of the ellipsoid (*x̄*, *ȳ*, *z̄*) and decreases in a distance-dependent manner according to the Gauss function. The mean value at which the Gauss function reaches its maximum is localized at the (0.0.0) point in a coordinate system. The standard deviation values σ*_x_*,σ*_y_*,σ*_z_* calculated for each dimension (axis) separately represent the size of the drop which depends on the length of the polypeptide under consideration [7].

The *j-th* grid point, for which the hydrophobicity is calculated, represents the effective atom position (averaged position of the side chain including Cα atoms) making possible attachment of a particular hydrophobicity density to a particular amino acid.

Before the external hydrophobic force field can be defined for any protein molecule, it shall be oriented in the space according to following procedure:

1. the geometric centre of the molecule shall be localized in the center of coordinate system.
2. the longest distance between two residues (represented by the effective atom – geometric centre of side chain of the amino acid) shall overlap one of the axes (say the X-axis).
3. the molecule shall be rotated around the X-axis to orient the longest inter-projections (on the YZ plane) distance along the Y-axis.
4. the linear size (the maximum inter-atomic distance along the X, Y, and Z axes) increased by 9 Å in each direction (the cutoff distance for hydrophobic interaction) makes possible calculation of σ*_x_*,σ*_y_*,σ*_z_*

This is how the geometric parameters of protein molecule can be interpreted according to the Gauss function.

Taking into account the high symmetry of the system under consideration, a user defined orientation of the coordinate system is necessary. Thus, the initial orientation determining the X-axis is defined by the position of the symmetrical units, so the averaged position of the top elements and averaged position of bottom elements (user-defined) determine the initial orientation of the molecule. The user-defined orientation of the molecule is available and necessary for any protein molecules or complexes before the “fuzzy oil drop” model can be applied. The X-axis defined this way is simultaneously the 7-fold symmetry axis.

The empirical (observed) distribution of hydrophobicity can be different than the idealized one. The empirical hydrophobicity distribution can be calculated according to Levitt function [16]:

$$\tilde{H}o_{j}=\frac{1}{\tilde{H}o_{sum}}\sum_{i=1}^{N}(H_{i}^{r}+H_{j}^{r})\{\begin{array}{l} \left[1-\frac{1}{2}\left(7{\left(\frac{r_{ij}}{c}\right)}^{2}-9{\left(\frac{r_{ij}}{c}\right)}^{4}+5{\left(\frac{r_{ij}}{c}\right)}^{6}-{\left(\frac{r_{ij}}{c}\right)}^{8}\right)\right]\\ 0\;\text{for}\;r_{ij}>c \end{array}\;\text{for}\;r_{ij}\leq c$$

where *Ho_j_* represents the empirical hydrophobicity value characteristic for the *j-th* grid point, $H_{i}^{r}$ represents the hydrophobicity characteristic of the *i*-th amino acid, *r_ij_* is the distance between the *j*-th grid point and *i*-th effective atom in the amino acid, and *c* expresses the cutoff distance, which has a fixed value of 9.0 Å following the original paper [16].

The continuity of the Gauss function allows calculation of the hydrophobicity density in any point in space (in the protein body). So any point can be treated as a grid point. It can also be the position of an effective atom in particular. This is why the index *j* may represent the position of *j-*th residue, as it is taken in this work. *Ho_sum_* represents the sum of all the grid points hydrophobicity. Any hydrophobicity scale may be applied to calculate the observed density of hydrophobicity [16–21].

Since both values are standardized (the coefficient $\frac{1}{H_{\text{sum}}}$) the differences between theoretical and empirical values expressing hydrophobicity density in a particular point of space (position of the side chain of *i-*th amino acid) can be calculated according to:

$$\Delta {\tilde{H}}_{i}=Ht_{i}-Ho_{i}$$

The profile of Δ*H̃_i_* (expressing the value of difference for each amino acid) reveals some maxima, which are related to hydrophobicity deficiency. The hydrophobicity deficiency (Δ*H̃_i_* > 0) seems to represent the potentially ligand binding site. The potential ligand may adhere in this area as the complementary element compensating the hydrophobicity deficiency producing in effect the regular smoothed hydrophobicity distribution. The values of negative Δ*H̃_i_* represent the area of hydrophobicity higher than expected. The area of such characteristics, when localized on the surface of protein seems to represent the potential area responsible for protein-protein complex creation.

The profile of Δ*H̃_i_* values can show the discrepancy between the idealized and empirical hydrophobicity density distribution revealing the fragments (or individual residues) representing the hydrophobicity excess (Δ*H̃_i_* negative) and hydrophobicity deficiency (Δ*H̃_i_* positive). It is expected that the hydrophobicity irregularity versus idealized one may express localization of biological function-related area in the protein body.

### 2.3. Protein Partitioning

The 1AON is a quite large and complex protein molecule. This is why different approaches have been applied.

1. The complete molecule was treated as one uniform “drop” – the orientation of molecule was according to its 7-fold symmetry axis (the X-axis).
2. The chaperonin molecule represents three levels organization: two stacked rings (Gro-EL) with the third one (Gro-ES) as “cap”.
3. Each ring (Gro-EL) and the “cap” (Gro-ES) is composed of seven identical polypeptide chains which are also treated as structural units (chains A-H, G-N and O-U).
4. The polypeptide chains belonging to Gro-EL evidently represent the two-domain construction. This is why each such domain is treated as independent individual part and treated as a “drop”.

**Figure 1.**
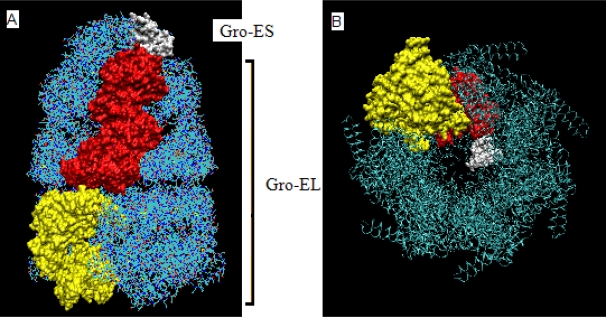
The chaperonin structure. A – Gro-ES (cap) built of the chains O-U – chain O distinguished in white, Gro-EL built of two rings: *cis* - chains A-G (chain A distinguished in red), *trans* – chains H-N (chain H in yellow); B – the axial view showing the seven-fold symmetry of the complex. The chains colored as in A.

The partitioning shown above is aimed to define the folding unit and possible path leading to complex generation (Figure 1). 1AON represents the chaperonin additionally complexed with ADP and some Mg^+2^ ions. The characteristics of localization of these ligands will be analyzed with respect to the construction of the “fuzzy-oil-drop”.

### 2.4. Identification of the Non-Bonding Interactions

The cut-off of 3.9 Å was taken to identify the residues interacting with ions, ligand (ADP) and protein (chain). The cut-off value has been taken according to the criteria applied in PDBsum [22] data base to make possible the comparison of results.

### 2.5. Implementation

All results have been obtained using our own program, written in Python [23] programming language. The program has been divided into multiple subroutines to ensure flexibility and diversity in dealing with PDB [24] files, which was required to work on protein partitions described above.

The first routine reads the input PDB file, removes all water residues and classifies non-empty chains into three groups: protein, nucleic and “hetero”. By “hetero” we mean neither protein nor nucleic acid chains, presumably containing only heteroatoms. All operations regarding PDB files are conducted using methods implemented in the Biopython [25] library.

The second routine is the core of the “fuzzy oil drop” evaluation: it performs an extraction of the residue subsets from the protein chains that the user is interested in and computes their effective atoms, which form the “drop”. The drop is placed in the origin and rotated to achieve a desired spatial orientation, as stated in the model description. After the drop size becomes known, theoretical and observed hydrophobicity are evaluated using optimized array operators from Numpy [26] (Numerical Python) library.

Identification of interactions is conducted using a fast k-d tree algorithm implemented in the Biopython library by checking position of each atom from selected residues against all atoms in file. Each neighbor atom placed within given radius of 3.9 Å is unfolded into parental residue and then classified as either protein, nucleic, ligand or ion contact. Contacts with protein residues from same chain are ignored.

The last routine sets output values for every residue (same for each atom) in PDB file: normalizes hydrophobicity discrepancy by overwriting the beta-factor column and contact type by overwriting the occupancy column. Plotting of graphical representation of results is made using the Matplotlib library [27].

### 2.6. Clustering Analysis

The agglomerative hierarchical clustering “ahc” algorithm has been applied to analyze the agreement between subjective interpretation of the “fuzzy oil drop” model applicability and objective discrimination of elements representing the common characteristics [28]. Clustering is the assignment of objects (feature vectors) into groups called clusters so that objects from the same cluster are more similar to each other than objects from different clusters. The short description of “ahc” algorithm is as follows. Suppose we have a data set *X* = {*x*_1_,..., *x_N_*} of *N* objects to be clustered and a user-defined distance measure *d*(*x_i_*, *x_j_*) to state how similar the two objects *x_i_* and *x_j_* are, for any *i* and *j*. The “ahc” algorithm starts off clustering this data by putting each of the data objects *x_i_* in a singleton cluster *C*_{*i*}_ = {*x_i_*} and then keeps on joining the closest pair of clusters *C*_{*i*}_ ∩ *C*_{*j*}_ = *C*_{__*i,j*}_ until there is only one large cluster *C*_{1,2,...,*N*}_. Simultaneously, the clustering tree (dendrogram) is built from leaves towards root, where merging of clusters is depicted as a common parent for two sub-trees. The distance between clusters *C_i_ = C*_{*i*_1_,...,*j*_*p*_}_ and *C_j_* = *C*_{*j*_1_,...,*j_r_*}_ can be measured in different ways. Three popular methods are single, complete and average linkage:

(single) $$\begin{array}{l} d_{\text{min}}\;(C_{i},C_{j})=\underset{x\in C_{i},y\in C_{j}}{\text{min}}d(x,y) \end{array}$$

(complete) $$\begin{array}{l} d_{\text{max}}\;(C_{i},C_{j})=\underset{x\in C_{i},y\in C_{j}}{\text{max}}d(x,y) \end{array}$$

(average) $$\begin{array}{l} d_{avg}(C_{i},C_{j})=\frac{1}{\left|C_{i}\right|\cdot \left|C_{j}\right|}\sum_{x\in C_{i},y\in C_{j}}d(x,y) \end{array}$$

where | *C* | denotes the number of objects in a cluster C. Thus, the distance between clusters is the minimum (or maximum or average) of the distances between one object from the first and another from the second cluster. Once the dendrogram is generated for assumed *k* clusters, the procedure cuts the *k-1* longest links in a dendrogram. The described “ahc*”* algorithm was implemented as a function in the software package Matlab v.7.

First, the implemented “ahc” algorithm was used to cluster separately objects in each of the files according to the partition (section 2.3) into *k=2* groups. Each object *x_i_* in these files is represented as a four dimensional feature vector *x_i_* = (*x*_*i*1_, *x*_*i*2_, *x*_*i*3_, *x*_*i*4_), where *x*_*i*1_, *x*_*i*2_, *x*_*i*3_ are the spatial coordinates *x,y,z* of the object *x_i_* while *x*_*i*4_ is its estimated variable Δ*H̃*. For each object *x_i_* its correct classification (being label ‘0’ or ‘1’ (i.e. “0” – means residue engaged in protein-protein interaction, ‘1’ denotes the residue not engaged in the protein-protein interaction) is known (according to PDBSum criteria). The clustering result for each partitioning form (as described in Section 2.3) compared with the correct classification allowed the calculation of the clustering performance defined as the number of objects correctly assigned to a groups divided by the total number of objects in a file.

## 3. Results

The results may be summarized as follows:

A molecule of high complexity like 1AON is difficult to describe in a simple way.

The partitioning described in Methods part was introduced.

The chains A-H appeared identical taking the Δ*H̃* profiles as the criterion (results not shown) as well as the chains G-N. The chains present in Gro-ES part also appeared to represent the identical Δ*H̃* profiles. This is why the chains A, G and O were taken to represent particular rings.

### 3.1. Hydrophobicity Density Irregularity in Chaperonin Molecule

**Figure 2.**
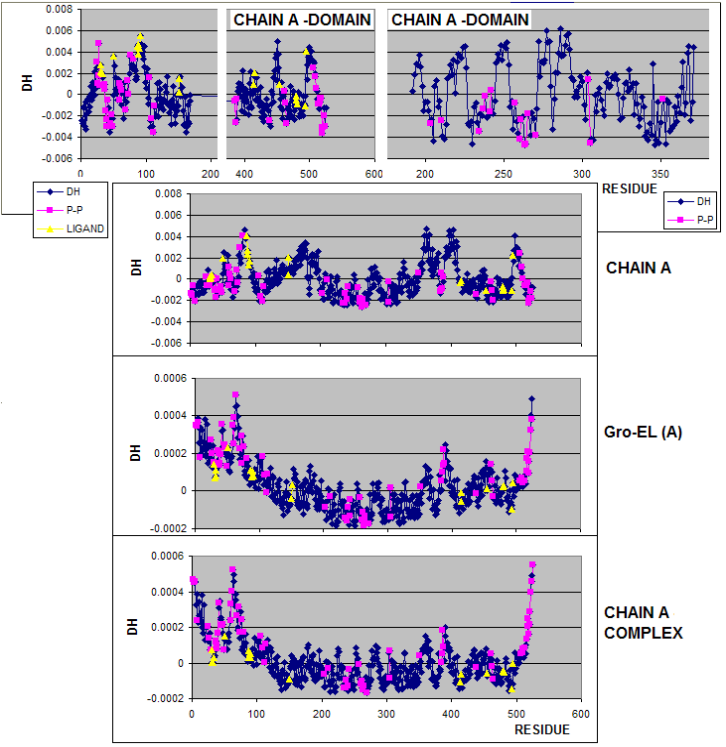
The Δ*H̃* profiles representing the irregularity of hydrophobicity density distribution in chain A calculated for units defined according to molecule partition: domains, chain, Gro-EL fragment and entire complex GroEL-GroES. The differences are the result of different orientation of chain A versus the oil drop definition. The yellow symbols represent the residues engaged in ligand binding, the pink symbols distinguish the residues engaged in protein-protein (P-P) interaction.

The Δ*H̃* profile of chain A taken to represent the chains A-G is shown in Figure 2. The residues engaged in ligand binding are distinguished as well as the residues involved in protein-protein complexation. According to “fuzzy oil drop” the Δ*H̃* minima representing the hydrophobicity excess on the surface of protein is assumed to be potential protein-protein contact area. The Δ*H̃* maxima (hydrophobicity deficiency) in domains distinguished in chain A are observed in accordance with the interpretation of “fuzzy oil drop” model being engaged in ligand (ADP) binding.

The residues of local Δ*H̃* minima are engaged in protein-protein interaction, particularly well seen in the domain 192–371. This accordance disappears step-wise taking the parts larger than the domain as the unit for drop construction localizing the ligand molecule even in the area of negative Δ*H̃* values. Both explanations for ligand binding are possible as the complementary accordance of target molecule and ligand. This observation is important for the interpretation of the “fuzzy oil drop” model.

Analysis of the Δ*H̃* profiles for chain A suggests that the construction of domains present in the chain A structure appears highly accordant to the interpretation of “fuzzy oil drop” model. The local Δ*H̃* minima are engaged in protein-protein complexation (particularly in the domain 192–371, while ligand molecule occupies the position of residues of local Δ*H̃* maxima. The characteristics of ligand binding residues in the Gro-EL and GroEl-ES complex get changed.

The characteristics of residues engaged in P-P or ligand binding changes due to different relative localization of these residues versus in the “oil drop” construction.

**Figure 3.**
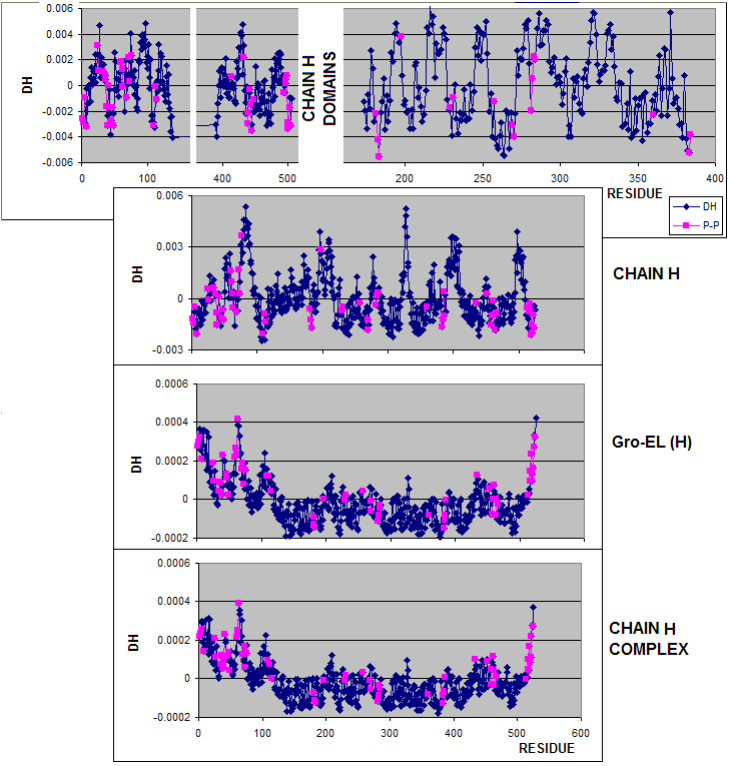
The Δ*H̃* profile representing the irregularity of hydrophobicity density distribution in chain H calculated for units defined according to molecule partition: domains, chain, Gro-EL fragment and entire complex GroEL-GroES. The differences are the result of different orientation of chain H versus the oil drop definition.

The same analysis performed for chain H representing the 7-fold system of the chains H-N is presented in Figure 3. There is no difference between the amino acids sequence in chains A-G and H-N, although some small differences of Δ*H̃* profiles are observed. It is the possible result of different complexation conditions (the chains H-N have no contact with the molecule Gro-ES) and no ligand is complexed to this ring.

The accordance of protein-protein interaction area with expectations based on “fuzzy oil drop” model is lower in chain H than in chain A.

**Figure 4.**
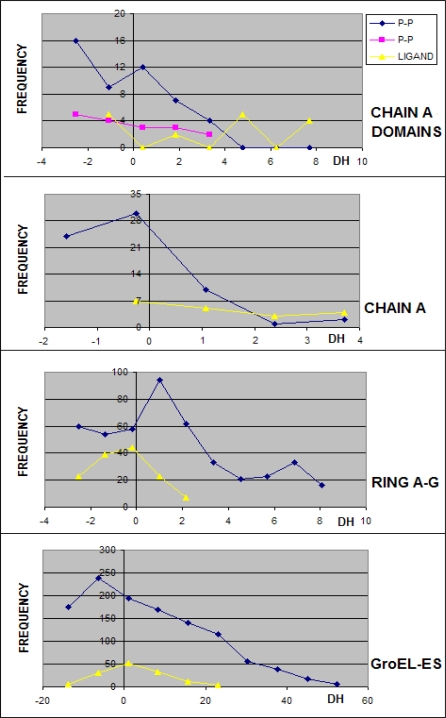
Frequency of the values of Δ*H̃* representing the residues in chain A engaged in protein-protein (P-P) interaction (the dark blue and pink curves – interaction of two domains) and in ligand complexation. The negative Δ*H̃* characterize the residues engaged in this type of interaction. Ligand complexation is represented by residues of positive Δ*H̃* values when the domain is taken as the unit.

**Figure 5.**
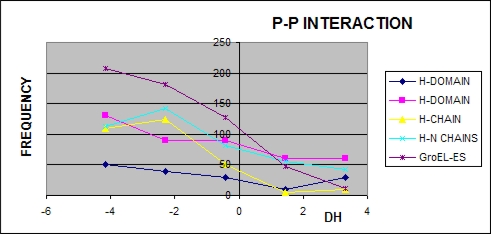
Frequency of the values of Δ*H̃* of residues in the chain H engaged in protein-protein (P-P) interaction. The negative Δ*H̃* are characteristic for this type of interaction.

According to expectations the residues involved in the protein-protein interaction represent the hydrophobicity excess (Δ*H̃* <0). The accordance is also found for the protein-ligand interaction which engages the residues of Δ*H̃*>0 hydrophobicity deficiency, which may be seen in Figures 4 and 5. The frequency of residues engaged in protein-protein complexation decreases together with the Δ*H̃* value (except the ring A-H which reaches the maximum for positive values of Δ*H̃*).

**Figure 6.**
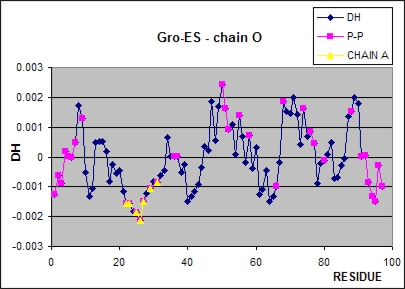
The Δ*H̃* profile for chain O as calculated for the entire Gro-ES fragment. The pink positions distinguish the residues engaged in protein-protein interaction, the yellow positions show the residues engaged in the interaction with the chain A of *cis* ring in the Gro-EL complex.

The Δ*H̃* profile of the chain O as calculated for the Gro-ES fragment reveals rather large fragments of hydrophobicity deficiency in this part of the chaperonin (Figure 6). The residues engaged in the interaction with the chain A are of special importance. Representing the very low values of Δ*H̃* (hydrophobicity excess) the residues of chain O fit well with the residues in chain A engaged in the interaction with this chain (residues 233-269), also representing the low values of Δ*H̃*. A high accordance can be seen in this case.

The residues of low Δ*H̃* values at the N- and C-terminal fragments are engaged in the interaction with chains of the *cis-*ring. This interaction seems to be of the form of hydrophobic interaction although the fragments of hydrophobic deficiency character are also engaged in protein-protein interaction.

In conclusion one may say that the H and A chain domains (especially the domain containing the residues 192 – 371) are highly accordant with the model. The domains generated by N-terminal and C-terminal polypeptide fragments seem also to be accordant with the “fuzzy oil drop” model.

### 3.2. Clustering

To make the interpretation of Δ*H̃* profiles more objective the clustering analysis was applied (as described in 2.6.) was performed. The results are shown in Table 1.

The highest accordance between expected (according to the “fuzzy oil drop” model) and observed (according to the PDBSum criteria) was obtained for the domain 192–371 in chain A. This observation supports the assumption that this domain could be the first one spontaneously folded according to “fuzzy oil drop” model exposing on the surface the hydrophobic residues in contact with other polypeptide chains. The chain H, although representing the identical sequence, displays some differences *versus* the A chain, suggesting it to be folded under other conditions than the chain A. Lower accordance between expected and observed classification of all other fragments of the complex (partitioning) suggest that the proper unit to be applied for protein folding in the environment simulated by the “fuzzy oil drop” is the domain 192–371 of the chain A. The relatively high accordance observed for the entire complex (chaperonin molecule) may be interpreted as the reliability of the “fuzzy oil drop” model. It suggests that the protein-protein interaction in the complete molecule may be recognized by the minima of Δ*H̃* in the profile. The results given in Table 1 seem to support the observations presented in Figures 4 and 5.

### 3.3. Structure-to-function Characteristics

The “fuzzy-oil-drop” model may be also applied for biological function recognition of the protein under consideration [10–12]. The role of chaperonin molecule (complex) is to create the environment for folding proteins. Thus, the internal surface characteristics of the capsule seems to be of special importance in respect to the biological function of this molecule.

**Figure 7.**
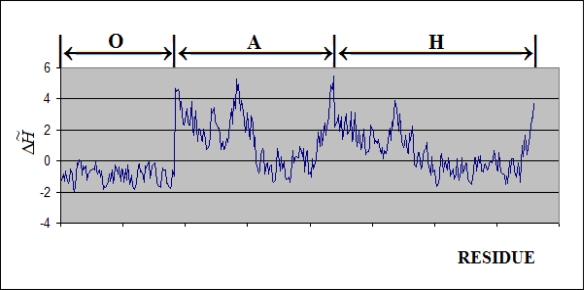
The Δ*H̃* profile of residues exposed on the internal surface ordered along the X-axis (7-fold symmetry). The Gro-ES (chains O – T) followed by the Gro-EL part: *cis-*ring generated by the chains A-G and the *trans-*ring created by the chains H-N.

**Figure 8.**
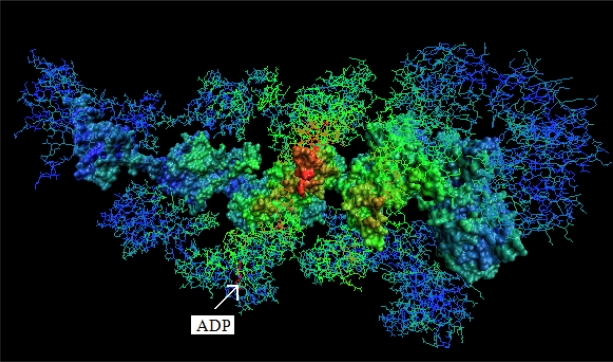
The O-A-H chain system (space filling presentation) in the company of two neighbors chains (T+P, B+G, I+N) in lines presentation. The color scale expresses the Δ*H̃* magnitude (the higher Δ*H̃* the more red color, the lower Δ*H̃* the more blue color). The green color represents the area of hydrophobicity accordant with the idealized ‘fuzzy oil drop” model. The molecule shown in red – the ADP molecule complexed to the protein.

The Δ*H̃* values of residues localized on the internal surface of the complex are presented in Figure 7 and the 3-D representation in Figure 8. These two figures show the residues ordered according to the X-axis localized on the internal surface of the capsule. The high excess of hydrophobicity in Gro-ES suggests the high participation of this type of interaction in substrate binding. The *cis*-ring (chains A-H), in contrast to the GroES part, presents highly differentiated characteristics expressing excess/deficiency hydrophobicity, although biased significantly toward the hydrophobicity deficiency. It may be interpreted as a specific distribution for stronger/weaker interactions with a substrate molecule. The *trans*-ring (H-N) presents rather low differentiation of hydrophobicity excess/deficiency with Δ*H̃* values close to zero (particularly in the end area). This suggests high accordance of the hydrophobicity distribution with the expected one (accordant with “fuzzy oil drop” model).

Figures 7 and 8 present the hydrophobicity irregularity of the internal channel where the folding reaction takes place. These two pictures show the characteristics of the external force field of hydrophobic character. Its specific deformation (in the sense of irregularity versus the idealized Gauss function distribution) seems to represent the localization of possible anchorage for folding protein in the chaperonin capsule.

**Figure 9.**
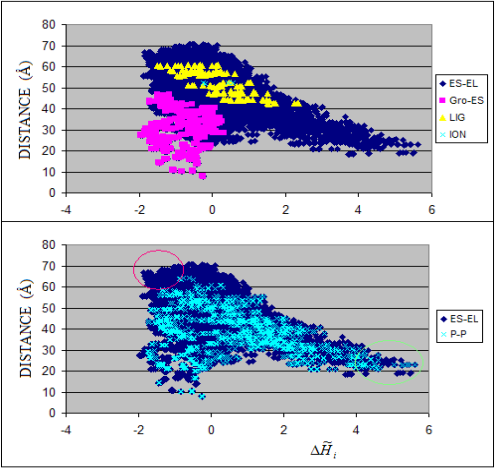
Relation between residue localization (distance) versus the 7-fold symmetry axis of residues and its Δ*H̃* values expressing the degree of hydrophobicity density irregularity. The dark blue symbols represent all residues present in Gro-EL Gro-ES complex. The pink symbols distinguish the residues of Gro-ES fragment. The yellow symbols show the characteristics of residues engaged in ligand binding. The light green symbols show resides engaged in ion binding. The light blue symbols in lower picture represent the residues engaged in protein-protein interaction. The residues in the area distinguished by red circle represent residues on the chaperonin surface potentially ready for hydrophobic protein-protein interaction. The residues (belonging to the set distinguished by green circle) localized on the internal cylinder surface of high Δ*H̃* are potentially ready (not being engaged in any other interaction in the complex) to interact according to non-bonding interaction category. P-P denotes the protein-protein contact, ES-EL – the entire complex, Gro-ES the fragment ES, LIG – interaction with ADP and ION – residues engaged in ion binding.

The discussion of hydrophobicity based structure–to-function relationships also concerns other parts of the complex presenting highly irregular hydrophobicity distributions. Particularly the excess hydrophobicity areas not engaged in protein-protein interactions (responsible for complex generation) seem to represent the regions potentially ready to interact under changed circumstances during the action of the chaperonin. The structural changes are reported to be a large deviation from the 7-fold symmetry of Gro-EL rings [14]. The possible protein-protein interaction can be simulated linking two hydrophobicity-excess areas of interacting chains. This can be seen in Figure 9.

The large–scale structural changes observed as accompanying the folding process engage residues potentially ready to interact. The residues localized on the surface (large distance versus the 7-fold symmetry axis) carrying highly negative Δ*H̃* values seem to be ready for hydrophobic interaction (distinguished by red circle in Figure 9). The residues localized closely versus the 7-fold symmetry axis representing large positive Δ*H̃* values are ready for non-bonding interaction (distinguished by green circle in Figure 9). The first possibility seems to be related to structural changes in the chaperonin molecule while the second one seems to be related to the interaction with the folding protein molecule (internal surface of the capsule) [29].

## 4. Conclusions

The “fuzzy oil drop” model was assumed to identify the area of excess hydrophobicity on the protein surface as a potential area for protein-protein interaction(s). The analysis of 1AON was treated as an example allowing the estimation of the limits of the applicability of this model for protein-protein interaction areas and ligand binding predictability. The CAPRI [30] initiative is oriented on protein-protein complexation blind prediction. The presented model was assumed to apply for protein-protein interaction recognition. According to the results shown in this paper, some fragments of polypeptide representing local Δ*H̃* minima in the profile can be treated as potential regions engaged in protein-protein complexation, particularly when calculated for domains present in the protein structure. The specificity of individual domains is able to determine the protein-protein complexation. They may be treated as the original source for this process. The ligand localization appeared also to be accordant with expectations – in the fragments of high Δ*H̃* values. Additionally the analysis of histograms particularly calculated of domains (which are assumed to be folded independently) perfectly well supports this interpretation (see Figure 4 and Figure 5.).

The fragments of low Δ*H̃* values not engaged in protein-protein interaction in the complex under consideration seem to represent fragments potentially ready for this type of interaction. The large structural deformations experimentally observed in chaperonin molecule during the protein (substrate) folding seem to be possible in molecule with hydrophobic areas on the protein surface, which is what is observed in 1AON [14].

This observation seems to be additionally supported by the well defined correlation between the hydrophobicity of a side-chain and the logarithm of the folding rate that has been reported in [31], where almost perfect linear correlation has been found for ΔΔG versus the change in hydrophobicity plots observed for few proteins. This is why the analysis of hydrophobicity distribution in protein bodies seems to be of high importance.

The “fuzzy oil drop” model was generated to represent the external force field to generate the environment for folding process assumed to direct the hydrophobic residues toward the center of the molecule and exposure of hydrophilic residues on the surface. The specific irregularity (Δ*H̃* profile) appeared to be biological function related [12]. This observation is assumed to support the postulated hypothesis of the necessary specific ligand participation in folding process to ensure the generation of highly specific cavity (ligand binding) [6–12].

The influence of external force field seems to be obvious in the case of the folding process assisted by a chaperonin molecule. This molecule is assumed to create the proper environment for folding polypeptide chains [31]. The hydrophobicity based characteristics of the interior of the capsule of chaperonin molecule seems to be able to direct the folding process in the form of controlled hydrophobicity excess/deficiency distribution in the folding molecule. The fragment of high positive Δ*H̃* (hydrophobicity deficiency) values fixes the non-bonding interactions and the fragments of low Δ*H̃* (hydrophobicity excess) constraints the hydrophobicity based interactions keeping the hydrophobic residues on the surface of the folding polypeptide if necessary. Assuming that the interior of the Gro-EL chamber really introduces the restraints of this character, the folded molecule shall represent the structure of Δ*H̃* distribution on the protein surface complementary to the internal surface of the chaperonin chamber (shown in Figures 8 and 9). This hypothesis is currently in the focus of the analysis.

## Figures and Tables

**Table 1.** Accordance between expected clustering and observed one for classification between amino acids engaged in protein-protein complexation versus all others (not engaged in protein-protein interaction) – third column and amino acids engaged in protein-protein complexation versus those which are not in protein-protein contact (the residues engaged in ligand or ion binding excluded). P-P denotes the residues engaged in protein-protein interaction.

PROTEIN	METHOD	ACCORDANCE	
		P-P versus all others	P-P versus not complexed
A-domain 1–191 371–524	HIERARCHY DMIN HIERARCHY DMAX HIERARCHY AVG	**0.8404** **0.7590** 0.6254	**0.8293** **0.7422** 0.5993
A-domain 192–371	HIERARCHY DMIN HIERARCHY DMAX HIERARCHY AVG	**0.9000** **0.7167** **0.7667**	**0.9000** **0.7167** **0.7667**
Chain A	HIERARCHY DMIN HIERARCHY DMAX HIERARCHY AVG	**0.8702** 0.5553 0.5668	**0.8651** 0.5377 0.5496
Chains ABCDEFG	HIERARCHY DMIN HIERARCHY DMAX HIERARCHY AVG	**0.8760** 0.5461 0.5774	**0.8710** 0.5445 0.5808
Chain O	HIERARCHY DMIN HIERARCHY DMAX HIERARCHY AVG	0.6598 0.5773 0.5979	0.6598 0.5773 0.5979
COMPLEX	HIERARCHY DMIN HIERARCHY DMAX HIERARCHY AVG	**0.8404** 0.5145 0.5062	**0.8554** 0.5069 0.5563
